# Chemical Fertilizer Reduction Combined with Microbial Fertilizer Improved Vegetation and Soil Characteristics in Degraded Alpine Meadows

**DOI:** 10.3390/plants15081174

**Published:** 2026-04-10

**Authors:** Yajuan Li, Lujie Li, Juan Du, Haiyan Li, Changlin Xu

**Affiliations:** College of Patacultural Science, Gansu Agricultural University, Key Laboratory of Grassland Ecosystem, Ministry of Education, Sino-U.S. Center for Grazing Land Ecosystem Sustainability, Pratacultural Engineering Laboratory of Gansu Province, Lanzhou 730070, China; 1073324120161@st.gsau.edu.cn (L.L.); 19944164709@163.com (J.D.); lhy730200@163.com (H.L.); 17839766921@163.com (C.X.)

**Keywords:** alpine meadow, degradation, chemical fertilizer, microbial fertilizer, biomass, soil nutrients

## Abstract

Alpine meadow degradation is a serious challenge for animal husbandry and ecosystem safety in the Qilian Mountain area, northwest China. Although some restoration methods have been used, fertilization practices still rely heavily on chemical fertilizers. As a type of green and effective fertilizer, microbial fertilizer was put into a degraded alpine meadow in this study, and six fertilization treatments, including no fertilization (CK), diammonium phosphate (600 kg∙ha^−1^) (DP), microbial fertilizer (75 kg·ha^−1^) (MF), diammonium phosphate (600 kg∙ha^−1^) with microbial fertilizer (75 kg·ha^−1^) (DPMF1), diammonium phosphate (450 kg∙ha^−1^) with microbial fertilizer (75 kg·ha^−1^) (DPMF2), and diammonium phosphate (300 kg∙ha^−1^) with microbial fertilizer (75 kg·ha^−1^) (DPMF3), were conducted on a moderately degraded alpine meadow using field plot experimental methods to evaluate the effects of reduced chemical fertilizer combined with microbial fertilizer on the vegetation and soil characteristics of degraded alpine meadow in 2023 and 2024. The results indicated that DP showed the highest biomass production in the two study years, but there was no significant difference between DPMF2 and DP in 2024. The dominance of originally fine forage *Kobresia humilis* and *Medicago ruthenica* showed the highest values for the DPMF3 treatment in 2023 and for the DPMF2 treatment in 2024. The vegetation Shannon–Wiener diversity and richness indices of DPMF1, DPMF2 and DPMF3 were significantly higher than those of CK. However, community diversity decreased in the second year of fertilization. DPMF2 treatment significantly increased the contents of soil organic matter, available nitrogen and available phosphorus in 2024. Grey correlation analysis indicated that 450 kg·ha^−1^ of diammonium phosphate combined with 75 kg·ha^−1^ of microbial fertilizer was the most suitable regime for moderately degraded alpine meadow restoration in the study area.

## 1. Introduction

There is widespread alpine meadow in Qilian Mountain, and it is an important base for local animal husbandry and a crucial ecological barrier in northwest China [1]. The unique geographical location and specific mountain climatic conditions of the area produce fragile habitats [2]. The regional alpine meadow has been seriously degraded in recent years because of unreasonable utilization and climate change [3]. The productivity of alpine meadow has been declining, which further aggravates the contradiction between forage and livestock amount and seriously affects the sustainable development of animal husbandry, the local ecological environment and the social economy [4]. Therefore, restoration of degraded alpine meadows is urgent.

Some studies have reported that restoration techniques, such as reasonable grazing, artificial grassland establishment, enclosure, no-tillage reseeding, ploughing and fertilization, have been proven to be effective for vegetation coverage and soil fertility improvement in degraded grassland [5,6]. In these techniques, fertilization has been frequently practiced for its faster and better recovery effects [7,8]. The long-term overuse of alpine grassland has led to serious soil available nutrient consumption and loss, which seriously restricts the self-recovery of degraded alpine meadow [9]. Therefore, it is necessary to add exogenous substances to supply soil nutrients for the restoration of degraded grasslands [10]. Reasonable fertilization can improve grassland plant composition [11], soil nutrient levels and soil microorganism characteristics, such as increasing soil microbial biomass, community structure and diversity [12,13], but the effect is related to fertilizer type, fertilizer amount, fertilization duration and habitat conditions [14]. A study indicated that the effect of nitrogen and phosphorus fertilizer on the aboveground biomass of grassland exceeded that of potassium fertilizer [15]. Although the extensive use of chemical fertilizers can rapidly promote the growth of forage in a short time in grassland [16], they are used with caution against the background of excessive application of chemical fertilizers in recent years in China. The soil environment in many areas has been destroyed, resulting in the deterioration of soil physical and chemical properties, soil acidification, and soil compaction because of the excessive application of chemical fertilizers, which limits the sustainable productivity and quality of vegetation [17]. Microbial fertilizer, as a functional fertilizer, has the advantages of non-toxicity, no pollution, low input and high yield which has been considered and applied in planting systems and even natural grassland systems [18,19]. Globally, replacing or partially replacing chemical fertilizers with new non-chemical fertilizer sources has become a new fertilization strategy in agricultural ecosystems [20].

As an active component of soil, microorganisms are sensitive to changes in soil nutrients. The degradation of alpine meadows has led to a decrease in soil microbial biomass [21]. In addition, soil microorganisms can produce inorganic nutrients such as nitrogen and phosphorus, which promote the process of grassland nutrient cycling and maintain the substance transformation and energy flow in soil, which is important to the stability of the soil ecosystem [22]. Therefore, microbial fertilizer was introduced into a degraded alpine meadow to explore whether microbial fertilizer could partially replace chemical fertilizer and to find a reasonable application amount of chemical fertilizer combined with microbial fertilizer for the restoration of degraded alpine under the special climatic conditions. We hypothesized that (1) chemical fertilizer reduction combined with microbial fertilizer could maintain the productivity and improve the vegetation diversity of degraded alpine meadow; (2) it could increase the soil key nutrient content of degraded alpine meadow; and (3) a suitable application regime can be found for chemical fertilizer combined with microbial fertilizer in the study area.

## 2. Results

### 2.1. The Alpine Meadow Productivity of Different Fertilization Treatments

#### 2.1.1. Average Plant Height

**Figure 1:**
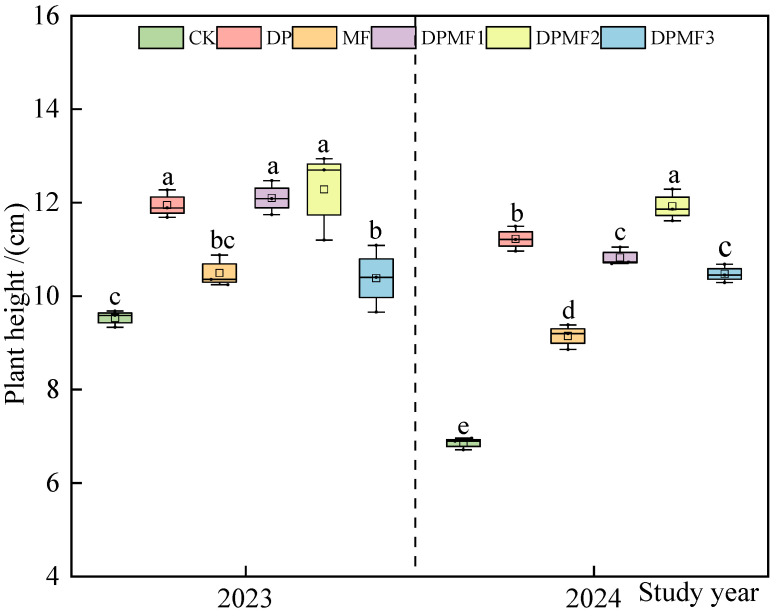
The average plant height under different fertilization treatments in 2023 and 2024. The different lowercase letters on the boxes in the figure indicate significant differences among different fertilization treatments at the 0.05 level; this is applicable for the following figures and tables as well.

The average plant heights of the vegetation community under the five fertilization treatments were significantly higher than those of CK in the two study years. The average plant height under DPMF1 was the highest in 2023, and there was no significant difference compared with DP and DPMF2. The average plant height under DPMF1 and DP treatments increased by 48.39% and 16.44%, respectively, compared with CK. The average height of the vegetation community under DPMF2 was the highest in 2024. The average height of the vegetation community under the DPMF2 and DP treatments was increased by 73.95% and 63.76%, respectively, compared with CK. Moreover, the height of the vegetation community under the DPMF1 treatment increased by 57.94% compared with CK. The average plant heights of the vegetation community under all treatments in 2024 were lower than those in 2023 (Figure 1).

#### 2.1.2. Aboveground Plant Biomass

**Figure 2:**
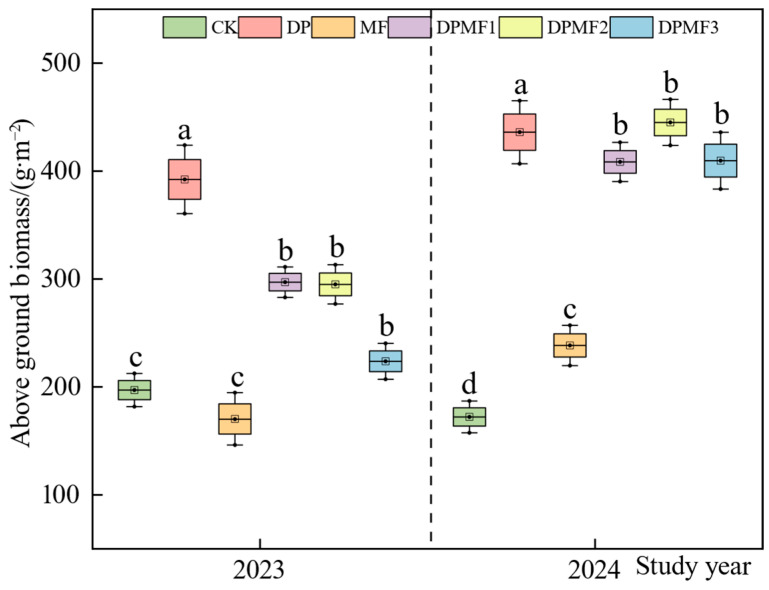
Aboveground plant biomass (dry weight) under different fertilization treatments in 2023 and 2024.

Almost all the fertilization treatments increased the aboveground dry grass weight of the degraded alpine meadows in two study years compared with CK, except for the MF treatment in 2023 (Figure 2). The aboveground dry grass weights under the DP treatment in 2023 and 2024 were both the highest, while they were 392.27 g∙m^−2^ in 2023 and 436.01 g∙m^−2^ in 2024. They were significantly different from those under the diammonium phosphate combined with microbial fertilizer treatments. There were no significant differences among the three diammonium phosphate combined with microbial fertilizer treatments, both in 2023 and 2024. But all five fertilization treatments showed higher aboveground plant biomass, while no fertilization treatment showed lower aboveground plant biomass in 2024 than in 2023. The MF treatment did not show a significant effect on aboveground biomass in the two study years (Figure 2).

### 2.2. The Vegetation Community Characteristics of Different Fertilization Treatments

#### 2.2.1. Plant Species Composition and Dominance

Although nineteen main plant species were found in all fertilization plots, the dominance of each species was changed under different fertilization treatments in the two study years, as shown in Table 1. *Kobresia humilis*, *Medicago ruthenica* and *Elymus nutans* were the original dominant plant species and fine forage for animal husbandry. The dominance values of *Kobresia humilis* under the MF, DP, DPMF1, DPMF2 and DPMF3 treatments were 38.0, 55.7, 58.7, 43.8 and 64.7, which increased by 46.97%, 0.18%, 54.74%, 10.05% and 61.19%, respectively, compared with CK in 2023. The dominance of *Medicago ruthenica* was lower in the CK and MF treatments, but it was increased under the three reduced diammonium phosphate and microbial fertilizer treatments. The dominance of both *Kobresia humilis* and *Medicago ruthenica* showed the highest level under the DPMF3 treatment in 2023 and the DPMF2 treatment in 2024. The highest dominance of *Elymus nutans* was 10.6% in 2023 and 29.4% under the DPMF3 treatment, and the three reduced diammonium phosphate and microbial fertilizer treatments increased the dominance of *Elymus nutans* sharply in 2024. The three key original dominant plant species showed higher dominance in 2024 than in 2023.

The plant species *Gentiana straminea*, *Oxytropis ochrocephala* and *Artemisia smithi* were the main poisonous weeds for grassland animals. The three reduced diammonium phosphate and microbial fertilizer treatments decreased the dominance of poisonous weeds in 2024, especially under the DPMF2 treatment. Therefore, fertilization increased the dominance of fine forage and decreased the dominance of inedible or poisonous weeds.

#### 2.2.2. Species Diversity Characteristics

The results of the species diversity indices of different fertilization treatments are shown in Table 2. The Shannon–Wiener index of the DP treatment was the highest both in 2023 and 2024, and was significantly higher than those of CK and the other fertilization treatments (*p* < 0.05). The Shannon–Wiener index in 2023 was lower than that in 2024. Fertilization had no significant effect on Pielou’s evenness index in 2023, but the DP treatment significantly increased the Pielou evenness index in 2024. Margalef’s richness index of the DP treatment was the highest, and was significantly higher than those of CK and MF (*p* < 0.05). The Simpson indices of the DPMF1 and DPMF3 treatments were significantly higher than that of CK in 2023 (*p* < 0.05), and there was no significant difference among the DP, MF and DPMF2 treatments and CK. But the Simpson indices under the DP and DPMF2 treatments were significantly higher than those of CK and MF (*p* < 0.05) in 2024. In total, DP and DPMF2 presented higher Margalef richness and Simpson indices in 2024, which showed that the vegetation communities of the two treatments had richer species and higher evenness. Meanwhile, the Shannon–Wiener and Simpson indices decreased in 2024 compared with 2023.

### 2.3. Soil Key Nutrient Contents Under Different Fertilization Treatments

#### 2.3.1. Soil Organic Matter

**Figure 3:**
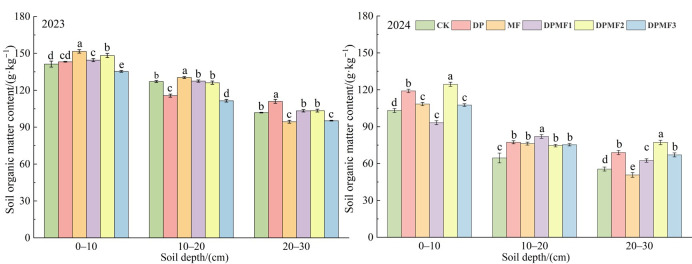
Soil organic matter contents under different fertilization treatments in the two study years.

The soil organic matter content under the MF treatment was significantly higher than that under the other fertilization treatments in 2023, which increased by 7.29% and 2.60% at 0–10 cm and 10–20 cm soil depths, respectively, compared with CK. But it was the highest under the DP treatment at the 20–30 cm soil depth and increased by 8.95% compared with CK in 2023 (Figure 3). The soil organic matter content under the DPMF2 treatment was the highest at the 0–10 cm soil depth, and increased by 20.63% and 4.54% compared with CK and DP at 0–10 cm, respectively, in 2024. It was significantly higher under DPMF1 than the other fertilization treatments at the 10–20 cm soil depth (*p* < 0.05), and increased by 27.09% and 6.11% compared with CK and DP, respectively, in 2024. Therefore, the diammonium phosphate combined with microbial fertilizer treatments promoted soil organic matter accumulation in the second fertilization year.

#### 2.3.2. Soil Total Nitrogen and Phosphorus Contents

**Figure 4:**
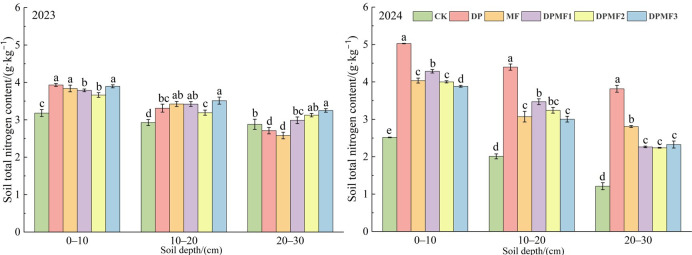
Soil total N contents under different fertilization treatments in the two study years.

All the fertilization treatments improved soil total nitrogen and phosphorus contents at both the 0–10 cm and 10–20 cm soil depth in the two study years compared with CK (Figure 4). There was no significant difference among DP, MF and DPMF3 treatments at 0–10 cm in 2023, but the order DP > DPMF1 > MF = DPMF2 > DPMF3 was shown in 2024. The soil total nitrogen content under the DP treatment was the highest at the three soil depths, and it was increased by 99.7%, 118.79% and 115.25% compared with CK at the 0–10 cm, 10–20 cm and 20–30 cm soil depths respectively. Meanwhile, the soil total nitrogen content under the CK treatment decreased in 2024 compared with 2023. An interesting result was that the soil total nitrogen content showed little difference between MF and the three chemical fertilizer and microbial fertilizer treatments at the 0–10 cm soil depth, both in 2023 and 2024 (Figure 4).

**Figure 5:**
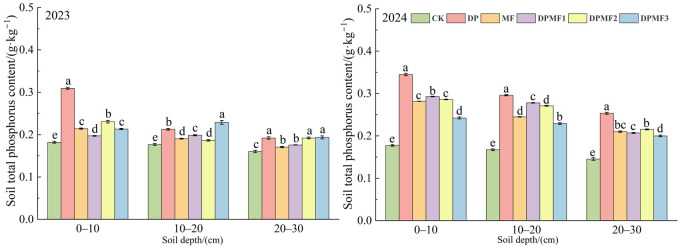
Soil total P contents under different fertilization treatments in the two study years.

The DP treatment showed the highest soil total phosphorus content at all soil depths in the two study years. The MF treatment and the three diammonium phosphate combined with microbial fertilizer treatments showed a weak positive effect on soil total phosphorus in 2023, but they greatly increased the soil total phosphorus content in 2024. The soil total phosphorus contents of MF, DPMF1, DPMF2 and DPMF3 increased by 58.75%, 65.06%, 61.17% and 36.51%, respectively, at the 0–10 cm soil depth compared with CK in 2024. The results also showed that the soil total phosphorus content of CK varied little between the study years of 2023 and 2024 (Figure 5). Therefore, the effect of the DP treatment on soil total nitrogen and phosphorus contents was better than that of DPMF1, DPMF2 and DPMF3.

#### 2.3.3. Soil Available Nitrogen and Phosphorus Contents

**Figure 6:**
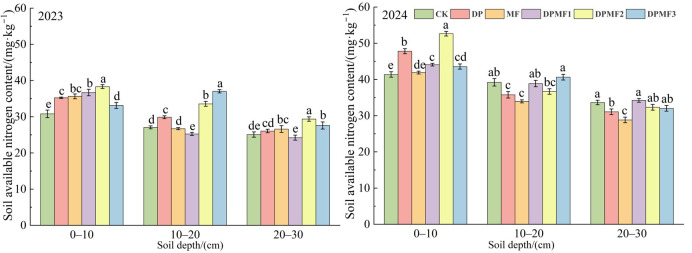
Soil available N contents under different fertilization treatments in the two study years.

**Figure 7:**
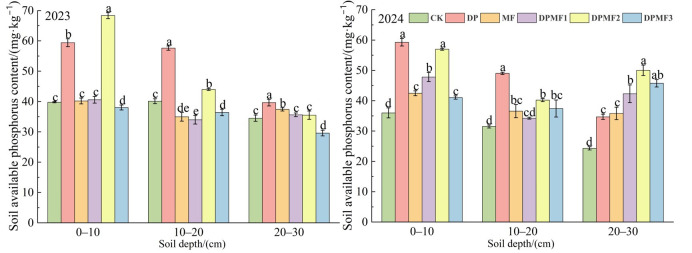
Soil available P contents under different fertilization treatments in the two study years.

All the fertilization treatments increased soil available nitrogen and phosphorus contents at the 0–10 cm soil depth in both study years (Figure 6). Both the soil available nitrogen content and the phosphorus content were the highest under the DPMF2 treatment at 0–10 cm, and increased by 24.43% and 27.22%, respectively, compared with CK for available nitrogen, and by 71.83% and 60.5% compared with CK for available phosphorus, in 2023 and 2024. The soil available nitrogen contents of all six treatments in 2023 were lower than in 2024, but the soil available phosphorus contents declined in 2024, which was not consistent with the changes in soil total nitrogen and phosphorus contents between the two study years. The soil available nitrogen content at the 10–20 cm soil depth under DPMF3 was the highest in both study years, and it was highest under the DPMF2 treatment in 2023, but it was highest under the DPMF1 treatment in 2024 (Figure 7). Therefore, the DPMF2 treatment effectively increased soil available nitrogen and phosphorus contents at the 0–10 cm soil depth in both study years.

#### 2.3.4. Grey Correlation Degree Analysis for Productivity and Soil Nutrient Indicators

The plant community height, aboveground biomass dry weight, soil organic matter, soil total nitrogen, soil total phosphorus, soil available nitrogen, and soil available phosphorus were selected to complete an integrated analysis for the six fertilization treatments using the grey correlation analysis method. The results showed that the rank of correlation degree for the six treatments was DP > DPMF2 > DPMF1 > MF > DPMF3 > CK in 2023 and DPMF2 > DP > DPMF1 > DPMF3 > MF > CK in 2024 (Table 3). The rank indicated that the DP and DPMF2 treatments presented well for the productivity and soil nutrient contents in the two study years. A single application of MF was not greatly beneficial for the restoration of the degraded alpine meadow.

## 3. Discussion

### 3.1. Effects of Fertilization on Degraded Alpine Meadow Productivity

The height of grassland vegetation reflects the utilization of light energy by plants and is also an important reflector of plant growth [23]. In this study, the plant height of the vegetation community under the fertilization treatments was significantly higher than that under the non-fertilization control, which indicated that all the fertilization treatments promoted plant growth in the degraded alpine meadow. This study showed that nitrogen and phosphorus addition significantly increased the plant height of the grassland community [24]. But there was no significant difference between the two study years in plant height, which was consistent with the result for the alpine Kobersia steppe meadow [25].

Changes in grassland biomass can greatly reflect the response of vegetation to various management practices [26]. This study showed that a single application of diammonium phosphate could improve soil nutrients quickly and directly, thus increasing aboveground biomass and community stability [27]. This study indicated that reduced chemical fertilizer combined with microbial fertilizer was beneficial to the aboveground biomass in the second year. Among the three diammonium phosphate with microbial fertilizer treatments, the aboveground biomass under the DPMF3 treatment was the highest in 2023, but there were no significant differences among them in 2024, which indicated that plants were not sensitive to the diammonium phosphate amount under the circumstance of microbial fertilizer addition. A single application of microbial fertilizer had no positive effect on aboveground biomass in the two study years, which proved that only microbial fertilizer was not useful for vegetation restoration in the degraded alpine meadow. Microbial fertilizer always shows a short-duration effect, and it responses differently among plant species. Meanwhile, low temperature has a more inhibitory effect on liquid microbial fertilizer but has little influence on chemical fertilizer [28]. As a liquid fertilizer was used in this study, the microbial fertilizer would definitely have been affected by the low temperature of the study area because the lowest temperature was 5 °C in the fertilization period. Therefore, the application of diammonium phosphate significantly increased the aboveground biomass of degraded alpine meadow, and reduced diammonium phosphate combined with microbial fertilizer improved the plant growth of the degraded alpine meadow after two years of fertilization.

### 3.2. Effects of Fertilization on Dominance and Species Diversity of Vegetation Community in Degraded Alpine Meadow

Plant species dominance, diversity and richness indices can reflect the ability of each species in the community to use resources and their ability to adapt to habitat conditions [29]. Fertilization always changes the growth of plants by changing the contents of nutrients in the soil, and then changes the plant community and brings a new round of vitality to the grassland [30]. The dominance results of plant species indicated that diammonium phosphate combined with microbial fertilizer improved the establishment of the fine forage community with the duration of fertilization, which may be related to the change in plant competitiveness after nutrient input in the meadow soil. Zi [31] pointed out that fertilization improved the availability of soil nutrients, changed the composition of grassland communities, and made the competition between plants change from underground to aboveground. Plant species did not change in the two study years, which may be because the fertilization time was not long. It was shown that long-term fertilization increased plant species [32]. Therefore, the effect of long-term fertilization should be investigated in this study area.

The species diversity index under the fertilization treatments increased compared with no fertilization, except for the MF treatment, while the evenness and richness indices did not change significantly. These results indicated that all the fertilization treatments did not significantly affect the stability of the community, which was consistent with the previous research [33]. This result proved that the addition of nitrogen and phosphorus did not increase the competition of plants for light in the alpine meadow community but increased the species diversity, maybe by affecting the elements required for the plant community [34,35]. It was found that genome size influences plant growth and biodiversity responses to nutrient fertilization in grassland [36]. Therefore, further studies can be conducted to explore the different responses of plant species to fertilization in the study area.

### 3.3. Effects of Fertilization on Soil Key Nutrients in Degraded Alpine Meadow

Soil organic matter mainly comes from the aboveground litter and underground roots in grassland. The amount of organic matter returned to soil always decreases gradually with the degradation degree of grassland [37]. Application of microbial fertilizer can increase soil organic matter and expand soil carbon and nitrogen supply levels, which can support forage yield and even alleviate the influence of climate change [38]. In this study, the organic matter content under the BM treatment was significantly higher than under the other treatments in 2023, and the soil organic matter content in the 0~10 cm soil layer under the DPMF2 treatment could reach 97.79% of that of BM, which highlighted that the microbial fertilizer played an important role in soil organic matter. However, the higher soil organic matter content under DP and DPMF2 in 2024 presented the advantage of chemical with microbial fertilizer for soil organic matter. Microbial fertilizers can aggravate the activity of soil microorganisms, promote the transformation of soil nutrients [39], and regulate the nutrients of rhizosphere soil, thus increasing soil organic matter levels [40]. Similarly, moderately reduced chemical fertilizer combined with microbial fertilizer can also maintain this advantage.

Soil available nutrients can be utilized directly by plants and are closely related to vegetation coverage [41]. Soil provides available nutrients that can be absorbed and utilized for plant growth and community development; meanwhile, the plant community can change soil physical and chemical properties [42]. In this study, the DPMF2 treatment effectively improved the soil available nitrogen and phosphorus supply level, and this is crucial to promote meadow restoration. The nitrogen-fixing and phosphate-solubilizing bacteria in microbial fertilizer can increase nitrogen and phosphorus availability. A study in semi-arid grassland ecosystems also showed long-term fertilization increased the soil nitrogen pool in grassland [43]. An appropriate combination of chemical and microbial fertilizers was a good fertilization regime for soil fertility restoration of the degraded alpine meadow.

## 4. Materials and Methods

### 4.1. Study Site

This study was conducted at the Alpine Grassland Experimental Station of Gansu Agricultural University, located in Zhuaxixiulong Town, Tianzhu County, Wuwei City, Gansu Province (37°10′16.97″ N, 102°47′17.31″ E). The study site consisted of a moderately degraded alpine meadow with 65% vegetation coverage. The average altitude is 2960 m, and the average annual evaporation is 1592 mm, while the annual precipitation is 416 mm, and most precipitation results from orographic lifting. The climate is cold and humid, the temperature difference between day and night is large, and solar radiation is strong during the daytime. There is no absolute frost-free period. The growing season lasts for up to five months per year. The soil type is mainly subalpine meadow soil and subalpine chernozem. The CaCO_3_ content at the 0–20 cm soil depth was 11.8%, the Ca^2+^ content was 152.4 mg.kg^−1^, the pH was 7.8, and the cation exchange capacity was 52.3 cmol.kg^−1^. The vegetation type is alpine meadow, mainly Poaceae and Cyperaceae plant species mixed with other forbs.

### 4.2. Experiment Design

A homogeneous area with similar habitat conditions, flat terrain, and uniform vegetation density was selected as the experimental area. The field experiment was conducted on the moderately degraded alpine meadow using a randomized plot experimental design. Six fertilization treatments were established: CK, DP (600 kg·ha^−1^), MF (75 kg·ha^−1^), DPMF1 (600 kg·ha^−1^ DP + 75 kg·ha^−1^ MF), DPMF2 (450 kg·ha^−1^ DP + 75 kg·ha^−1^ MF), and DPMF3 (300 kg·ha^−1^ DP + 75 kg·ha^−1^ MF). The six treatments and corresponding fertilizer application design are shown in Table 4. The application amount of the DP treatment was set according to the previous study by the authors’ group [44], and the application amounts of the MF treatment in all treatments were according to recommendations from the microbial fertilizer (MF) development team at our institution, who provided the MF product. The strains in MF included *Bacillus subtilis* (Numbered GAU-00667), *Bacillus mojavensis* (Numbered GAU-00660) and *Pseudomonas synxantha* (Numbered GAU-00668). The strains were cultured using LB medium. The viable count of MF was at least 1 × 10^9^ CFU/mL, and the pH was 7.5. MF, and it was stored under 15 °C conditions, avoiding high temperatures or freezing. The experiment used a randomized plot design. Each treatment had three replicates and 18 plots in total. Each plot measured 3 × 5 m (15 m^2^), and 0.5 m buffer strips were set between two plots. Diammonium phosphate (18% N; 46% P_2_O_5_) was purchased from the local agriculture market. Fertilization was conducted in early June when the grass was regreening in 2023 and 2024. Diammonium phosphate was evenly applied to the surface according to the amount of fertilizer applied in each plot, and then the microbial fertilizer was diluted using distilled water (the ratio of the microbial suspension to distilled water was 1:5) and applied evenly using a handheld sprayer. Fertilization was conducted on cloudy or light rainy days to ensure the effect of the bacterial fertilizer, and the CK treatment was applied with the same amount of distilled water to ensure the same soil moisture in each treatment.

### 4.3. Measuring Methods

#### 4.3.1. Vegetation Characteristics

The vegetation investigation was conducted in late August 2023 and 2024 (it was the early generative stage of most plants) using quadrat frame survey methods. The quadrat frame was a square, whose area was 0.25 m^2^ (0.5 m × 0.5 m), and three quadrats were randomly placed in each experimental plot. The plant species, plant height, plant coverage, and density in each quadrat frame were measured and recorded. The plants in each quadrat frame were cut at ground level, and the fresh weight of the grass was determined immediately after cutting in the field and then packed into paper bags and brought back to the laboratory to measure the dry weight. The data from every experimental plot were the averages of the three quadrat frames. The measurement details of the vegetation characteristics were as follows.

The total coverage and species coverage in the quadrat frame were measured using a needle-punched process: a needle of a 2 mm diameter was vertically passed through the grass layer, the name and frequency of every plant that the needle pricked was recorded, and the needle was moved every 5 cm for the next punch. The grass of different plant species in each quadrat frame was cut off from the soil surface, and the fresh grass was weighed. The accumulation of each plant species’ weight was the total fresh weight of aboveground biomass for each experimental plot. Then, the fresh grass was packed into a paper bag and transported to the lab quickly. Each grass sample was oven-dried at 105 °C for 30 min to halt enzymatic activity, and then it was dried at 65 °C until reaching a constant weight to get the dry weight of each plant species. The dry weight of all plants in each plot was the total dry weight of the aboveground biomass.

Ten plants in a plot were randomly selected to measure the natural height from the ground to the highest point of the plant, and the mean of the ten measurements was used as the plot-level plant height.

Meanwhile, the vegetation community structure and diversity of each plot were investigated and calculated. Dominance was calculated by the relative coverage, biomass and plant height of the same plant in all quadrat frames.

Dominance = (relative coverage + relative biomass + relative height)/3

Relative coverage=c/C

Relative boimass=m/M

Relative height=h/H

In the formulas above, c is the total number of needle contacts for each plant species, C is the total number of needle contacts for all species, m is the fresh biomass of each plant species, M is the total fresh biomass of one quadrat frame, h is the average height for each plant species, and H is the average plant height of all plant species in the quadrat frame.

The Simpson index (D), the Shannon–Wiener index (H), the Pielou evenness index (E), and the Margalef richness index (M) were calculated by following the formulae [45,46,47,48].

$$D=1-{\sum Pi}^{2}$$

$$H=-\sum \left(Pi\right)\left(\ln Pi\right)$$

E=H/lnS

$$M=\left(S-1\right)/\mathrm{lnN}$$

Pi represents the proportion of the biomass of the i specie in the community to the total biomass of the community, S is the number of species, and N is the number of individuals of all plant species in one quadrat frame.

#### 4.3.2. Soil Sample Collection and Measurement

Soil samples were collected randomly in every plot from 0–10 cm, 10–20 cm, and 20–30 cm depths, respectively, using a soil drill with a diameter of 3.5 cm after the vegetation community investigation and plant sampling. Five cores per depth were composited into a single sample. Each soil sample was put into a plastic self-sealing bag after picking out the stones and plant roots, and brought back to the laboratory as soon as possible. Then, the soil samples were air-dried and sieved through 1 mm and 0.25 mm sieves respectively. A 1 mm soil sample was used for the measurement of soil organic matter, total nitrogen and phosphorus, while a 0.25 mm sample was used for soil available nitrogen and phosphorus. The soil total nitrogen content was determined using the Kjeldahl method; total phosphorus was determined using ammonium molybdate coloration and the blue colorimetric method; soil total potassium was determined using the NaOH melting–flame photometer method; soil available nitrogen was determined using the alkali solution diffusion method; soil available phosphorus was determined using sodium bicarbonate (NaHCO_3_) extraction, ammonium molybdate coloration and the blue colorimetric method; soil available potassium was determined using the NH_4_Ac extraction–flame photometer method; and soil organic matter was determined using the potassium dichromate oxidation–external heating method [49].

#### 4.3.3. Grey Correlation Analysis

The measured data of grassland productivity and soil nutrient indicators were used to do the gray correlation analysis to evaluate the comprehensive effects of the fertilization treatments. Dimensionless treatment used the formula: ${X’}_{i}\left(k\right)=X_{i}\left(k\right)/X_{0}\left(k\right)$, *X* represents the tested treatments, *k* represents the indicators, and *Xi* is the comparison series composed of the measured values of *X* at index *k* under different fertilization treatments. The optimal values of each index of the different fertilization treatments are used as the $X_{0}$ best.

Relationship coefficient: $\varepsilon_{i}\left(k\right)=\frac{{min}_{i}{min}_{k}{∆}_{i}\left(k\right)+\rho {max}_{i}{max}_{k}{∆}_{i}\left(k\right)}{{∆}_{i}\left(k\right)+\rho {max}_{i}{max}_{k}{∆}_{i}\left(k\right)}$

Absolute difference: For the resolution factor, the value is taken between [0, 1], and the value in this study is 0.5. ${∆}_{i}\left(k\right)=\left|X_{0}\left(k\right)-X_{i}\left(k\right)\right|\rho$

Relevance: $\gamma_{i}=\frac{1}{n}\sum_{k=1}^{n}\varepsilon_{i}\left(k\right)$;

Weight coefficient: $\omega_{i}\left(k\right)=\frac{\gamma_{i}}{\sum \gamma_{i}}$;

Weighted correlation: ${\gamma ’}_{i}=\sum_{k\;=\;1}^{n}\omega_{i}\left(k\right)\varepsilon_{i}\left(k\right)$.

According to the principle of gray correlation analysis, the greater the correlation, the better its comprehensive evaluation performance [50].

### 4.4. Data Analysis

Excel 2017 was used to organize the result data, and SPSS 24.0 (SPSS statistical package, Chicago, IL, USA) was used to check the normality and homogeneity of variance and perform a one-way ANOVA analysis and a Duncan multiple comparison test on the data among the six fertilization treatments. None of the variables were transformed. The significance level was set at *p* < 0.05, and the data were expressed as means ± standard deviation. All the figures were created using Origin Pro 2022.

## 5. Conclusions

Reduced diammonium phosphate combined with microbial fertilizer significantly increased the vegetation community height and total aboveground biomass of the degraded alpine meadow and increased the dominance of edible grasses such as *Kobresia humilis* and *Medicago ruthenica* in the alpine meadow. Although DP presented the highest production, DPMF1 and DPMF2 showed stable production in the two study years, and the aboveground biomass was 406.1 g∙m^−2^ in 2024. The dominance of fine forage *Kobresia humilis* and *Medicago ruthenica* showed the highest values under the DPMF3 treatment in 2023 and under the DPMF2 treatment in 2024. The vegetation Shannon–Wiener diversity index and richness of diammonium phosphate combined with microbial fertilizer were significantly higher than those of CK. However, the diversity of alpine meadow communities decreased in the second study year. All the treatments of nitrogen and phosphorus fertilizer combined with microbial fertilizer increased the soil available nutrients and total nutrients. DPMF2 significantly increased the contents of soil organic matter, available nitrogen and phosphorus in 2024; particularly, the soil organic matter and available nitrogen contents were 4.5% and 10.1% higher than those under DP in the 0–10 cm soil. Grey correlation analysis indicated that DPMF2 (450 kg∙ ha^−1^ of diammonium phosphate combined with 75 kg∙ha^−1^ of microbial fertilizer) was a suitable fertilization regime for moderately degraded alpine meadow restoration in the Eastern Qilian Mountains, northwest China. Considering the short duration of this study, long-term effects and their mechanisms, such as the response of soil microorganisms to fertilization, are needed to further investigate in the future.

## Figures and Tables

**Table 1:** Species composition and dominance of alpine meadow plant communities under different fertilization treatments in 2023 and 2024.

Study Years	Plant Species	Dominance Value (%)					
		CK	DP	MF	DPMF1	DPMF2	DPMF3
2023	*Kobresia humilis*	37.9	55.7	38.0	58.7	43.8	64.7
	*Medicago ruthenica*	26.2	44.0	19.6	39.4	40.8	57.2
	*Agropyron mongolicum*	26.0	36.5	27.5	33.0	27.7	28.2
	*Elymus nutans*	3.8	9.5	1.0	4.3	4.9	10.6
	*Potentilla multicaulis*	2.6	4.6	3.0	4.5	2.9	4.6
	*Astragalus polycladus*	3.8	6.6	3.6	2.3	<0.5	1.0
	*Potentilla bifurca*	0.7	0.5	1.0	1.0	2.8	1.7
	*Potentilla discolor*	8.2	8.8	15.7	13.2	10.5	15.0
	*Aster tataricus.*	1.5	9.6	4.0	3.8	11.0	4.1
	*Oxytropis ochrocephala*	7.1	10.1	8.1	13.8	9.4	11.2
	*Allium sikkimense*	19.3	46.0	29.0	51.6	23.1	33.1
	*Gentiana straminea*	0.0	<0.5	1.4	1.5	0.5	0.5
	*Plantago depressa*	0.0	2.3	<0.5	0.7	<0.5	2.2
	*Taraxacum mongolicum*	0.5	0.6	<0.5	<0.5	1.0	<0.5
	*Artemisia smithi*	25.0	35.3	27.5	51.7	49.5	25.5
	*Thalictrum alpinum*	0.0	0.67	2.17	0.0	0.0	<0.5
	*Poa pratensis*	1.2	4.7	19.1	25.1	31.3	13.7
	*Stipa capillata*	8.8	10.4	8.5	12.1	12.3	10.2
	*Anaphalis lactea* Maxim.	2.3	7.1	5.2	10.0	8.1	12.4
2024	*Kobresia humilis*	41.4	71.9	54.9	69.4	79.4	64.4
	*Medicago ruthenica*	27.5	26.7	21.1	27.4	51.4	44.2
	*Agropyronmongolicum*	14.3	15.3	8.5	3.0	3.0	19.3
	*Elymus nutans*	1.0	15.8	10.7	26.2	32.1	29.4
	*Potentilla multicaulis*	7.2	0.0	0.9	3.6	<0.5	16.2
	*Astragalus polycladus*	2.1	0.0	1.0	6.2	3.6	6.3
	*Potentilla bifurca*	4.5	3.4	5.1	7.1	6.2	5.0
	*Potentilla discolor*	0.5	0.5	3.9	0.9	7.2	6.9
	*Aster tataricus.*	6.3	4.1	4.7	10.2	0.9	6.6
	*Oxytropis ochrocephala*	4.3	1.0	13.2	11.2	0.0	0.0
	*Allium sikkimense*	5.1	6.5	8.9	10.0	10.2	4.9
	*Gentiana straminea*	23.6	19.6	23.1	12.7	11.2	20.9
	*Plantago depressa*	1.9	9.5	0.0	7.9	<0.5	4.7
	*Taraxacum mongolicum*	3.7	0	<0.5	0	10.0	1.9
	*Artemisia smithi*	25.1	67.2	32.2	26.3	12.7	19.5
	*Thalictrum alpinum*	19.2	10.3	5.4	8.0	0	2.8
	*Poa pratensis*	10.0	35.5	14.5	5.8	26.3	7.1
	*Stipa capillata*	10.4	23.2	14.3	19.9	8.7	13.4
	*Anaphalis lactea* Maxim.	0	2.6	4.40	8.7	7.9	0

**Table 2:** Plant species diversity indices of different fertilization treatments.

Year	Fertilization Treatment	Shannon–Wiener Index	Pielou Evenness Index	Margalef Richness Index	Simpson Index
2023	CK	2.40 ± 0.07 c	0.85 ± 0.07 a	1.55 ± 0.26 c	0.75 ± 0.03 b
	DP	3.24 ± 0.09 a	0.84 ± 0.03 a	2.24 ± 0.27 a	0.78 ± 0.03 ab
	MF	2.48 ± 0.09 c	0.80 ± 0.09 b	1.48 ± 0.13 c	0.79 ± 0.05 ab
	DPMF1	3.10 ± 0.02 ab	0.86 ± 0.03 a	1.90 ± 0.10 ab	0.84 ± 0.02 a
	DPMF2	2.70 ± 0.17 bc	0.86 ± 0.14 a	1.70 ± 0.09 b	0.79 ± 0.05 ab
	DPMF3	2.76 ± 0.24 bc	0.87 ± 0.06 a	1.75 ± 0.09 b	0.85 ± 0.05 a
2024	CK	2.09 ± 0.04 b	0.82 ± 0.01 c	1.76 ± 0.16 b	0.40 ± 0.00 bc
	DP	2.84 ± 0.05 a	0.88 ± 0.02 a	2.14 ± 0.24 a	0.51 ± 0.00 a
	MF	2.59 ± 0.04 b	0.84 ± 0.02 bc	1.86 ± 0.24 b	0.37 ± 0.00 bc
	DPMF1	1.99 ± 0.05 c	0.83 ± 0.01 bc	1.54 ± 0.13 c	0.30 ± 0.01 c
	DPMF2	1.94 ± 0.06 c	0.86 ± 0.04 a	1.83 ± 0.28 b	0.46 ± 0.01 ab
	DPMF3	2.48 ± 0.30 b	0.85 ± 0.01 a	1.59 ± 0.14 c	0.35 ± 0.01 c

**Table 3:** Grey correlation degrees of different fertilization treatments.

Fertilization Treatment	2023		2024	
	Correlative Degree	Rank	Correlative Degree	Rank
CK	0.854	6	0.845	6
DP	0.975	1	0.973	2
MF	0.870	4	0.867	5
DPMF1	0.894	3	0.922	3
DPMF2	0.929	2	0.996	1
DPMF3	0.857	5	0.922	4

**Table 4:** Fertilization treatments s.

Treatment	Fertilization Regime
CK	No fertilization
DP	Diammonium phosphate 600 kg∙ha^−1^ (100% chemical fertilizer)
MF	Microbial fertilizer 75 kg∙ha^−1^ (100% microbial fertilizer)
DPMF1	Diammonium phosphate 600 kg∙ha^−1^ + microbial fertilizer 75 kg∙ha^−1^ (100% chemical fertilizer + microbial fertilizer)
DPMF2	Diammonium phosphate 450 kg∙ha^−1^ + microbial fertilizer 75 kg∙ha^−1^ (75% chemical fertilizer + microbial fertilizer)
DPMF3	Diammonium phosphate 300 kg∙ha^−1^ + microbial fertilizer 75 kg∙ha^−1^ (50% chemical fertilizer + microbial fertilizer)

## Data Availability

All data are contained within this article. Further inquiries can be directed to the corresponding authors.

## References

[1] Li W., Cao W., Wang J., Li X., Xu C., Shi S. (2017). Effects of grazing regime on vegetation structure, productivity, soil quality, carbon and nitrogen storage of alpine meadow on the Qinghai-Tibetan Plateau. Ecol. Eng..

[2] Zhang Y., Zhao W. (2015). Vegetation and soil property response of short time fencing in temperate desert of the Hexi Corridor, northwestern China. CATENA.

[3] Peng Q., Wang R., Jiang Y., Li C. (2021). Contributions of climate change and human activities to vegetation dynamics in Qilian Mountain National Park, northwest China. Glob. Ecol. Conserv..

[4] Li Y.-Y., Dong S.-K., Wen L., Wang X.-X., Wu Y. (2014). Soil carbon and nitrogen pools and their relationship to plant and soil dynamics of degraded and artificially restored grasslands of the Qinghai–Tibetan plateau. Geoderma.

[5] Liu J., Yang X., Ghanizadeh H., Guo Q., Fan Y., Zhang B., Yan X., Wen Z., Li W. (2021). Long-term enclosure can benefit grassland community stability on the Loess Plateau of China. Sustainability.

[6] Tai X., Epstein H.E., Li B. (2021). Effects of grazing exclusion on spring and autumn pastures in arid regions of China: Insights from field surveys and land sat images. Agric. Ecosyst. Environ..

[7] Bardgett R.D., Bullock J.M., Lavorel S., Manning P., Schaffner U., Ostle N., Chomel M., Durigan G., Fry E.L., Johnson D. (2021). Combatting global grassland degradation. Nat. Rev. Earth Environ..

[8] Chen W., Zhou H., Wu Y., Wang J., Zhao Z., Li Y., Qiao L., Chen K., Liu G., Xue S. (2020). Direct and indirect influences of long-term fertilization on microbial carbon and nitrogen cycles in an alpine grassland. Soil Biol. Biochem..

[9] Han J.G., Zhang Y.J., Wang C.J., Bai W.M., Wang Y.R., Han G.D., Li L.H. (2008). Rangeland degradation and restoration management in China. Rangel. J..

[10] Vuckovic S., Cupina B., Simic A., Prodanovic S., Zivanovic T. (2006). Effect of nitrogen fertilization and under sowing on yield and quality of Cynosuretumcristati-type meadows in hilly-mountainous grasslands in Serbia. J. Cent. Eur. Agric..

[11] Dong S., Shang Z., Gao J., Boone R.B. (2020). Enhancing the ecological services of the Qinghai-Tibetan Plateau’s grasslands through sustainable restoration and management in era of global change. Agric. Ecosyst. Environ..

[12] Kholod N., Evans M., Gusev E., Yu S., Malyshev V., Tretyakova S., Barinov A. (2016). Environmental and economic benefits of variable rate nitrogen fertilization in a nitrate vulnerable zone. Sci. Total Environ..

[13] He S., Du J., Wang Y., Cui L., Liu W., Xiao Y., Ran Q., Li L., Zhang Z., Tang L. (2024). Differences in background environment and fertilization method mediate plant response to nitrogen fertilization in alpine grasslands on the Qinghai-Tibetan Plateau. Sci. Total Environ..

[14] Gallego-Zamorano J., Huijbregts M.A., Schipper A.M. (2022). Changes in plant species richness due to land use and nitrogen deposition across the globe. Divers. Distrib..

[15] Zhang Z., Zhang X., Mahamood, Zhang S., Huang S., Liang W. (2016). Effect of long-term combined application of organic and inorganic fertilizers on soil nematode communities within aggregates. Sci. Rep..

[16] Eastwood C., Rue B.D., Kerslake J. (2020). Increases in forage legume biomass as a response to nitrogen input depend on temperature, soil characters and planting system: A meta-analysis. Grass Forage Sci..

[17] Li F., Minggagud H., Jarvie S., Wang Y., Yan Y., Gong X., Han P., Zhang Q. (2023). Mowing mitigates the adverse effects of fertilization on plant diversity and changes soil bacterial and fungal community structure in the Inner Mongolia grassland. Agric. Ecosyst. Environ..

[18] Yu Y., Zhang L., Li Y., Hou L., Yang H., Shi G. (2022). Silicon fertilizer and microbial agents changed the bacterial community in the consecutive replant soil of lilies. Agronomy.

[19] Shang X., Fu S., Guo X., Sun Z., Liu F., Chen Q., Yu T., Gao Y., Zhang L., Yang L. (2023). Plant growth-promoting rhizobacteria microbial fertilizer changes soils’ microbial structure and promotes healthy growth of cigar tobacco plants. Agronomy.

[20] Kai T., Nishimori S., Tamaki M. (2020). Effect of organic and chemical fertilizer application on growth, yield, and quality of small-sized tomatoes. J. Agric. Chem. Environ..

[21] Yang Y., Li M., Wu J., Pan X., Gao C., Tang D.W.S. (2022). Impact of combining long term subsoiling and organic fertilizer on soil microbial biomass carbon and nitrogen, soil enzyme activity, and water use of winter wheat. Front. Plant Sci..

[22] Kuypers M.M.M., Marchant H.K., Kartal B. (2018). The microbial nitrogen-cycling network. Nat. Rev. Microbiol..

[23] Wang Z., Li T., Wang C., Dang N., Wang R., Jiang Y., Wang H., Li H. (2021). N and P fertilization enhanced carbon decomposition function by shifting microbes towards an r-selected community in meadow grassland soils. Ecol. Indic..

[24] Zong N., Shi P., Zheng L., Zhou T., Cong N., Hou G., Song M., Tian J., Zhang X., Zhu J. (2021). Restoration effects of fertilization and grazing exclusion on different degraded alpine grasslands: Evidence from a 10-year experiment. Ecol. Eng..

[25] Wang W., Wang Q., Wang H. (2006). The effect of land management on plant community composition, species diversity, and productivity of alpine Kobersia steppe meadow. Ecol. Res..

[26] Sammul M. (2010). Length of the spacer rather than its plasticity relates to species distribution in various natural habitats. Folia Geobot..

[27] Hautier Y., Tilman D., Isbell F., Seabloom E.W., Borer E.T., Reich P.B. (2015). Anthropogenic environmental changes affect ecosystem stability via biodiversity. Science.

[28] Zhang L., Yuan F., Bai J., Duan H., Gu X., Hou L., Huang Y., Yang M., He J.-S., Zhang Z. (2020). Grassland responses to fertilization with nitrogen and phosphorus: A global review. Ecol. Lett..

[29] Gill J., Sale P., Peries R., Tang C. (2009). Changes in soil physical properties and crop root growth in dense sodic subsoil following incorporation of organic amendments. Field Crop. Res..

[30] Bai Y., Dai J., Wei Z., Cui Q., Zhang D., Qi X., Zhao Y., Liu H. (2025). Effects of nitrogen addition on soil nutrients and stoichiometry in desert steppe, Northern China. Appl. Ecol. Environ. Res..

[31] Zi H., Hu L., Wang C. (2022). Differentiate responses of soil microbial community and enzyme activities to nitrogen and phosphorus addition rates in an alpine meadow. Front. Plant Sci..

[32] Karel P., Kamila V., Klara R., Josef K. (2021). Three decades of vegetation changes in a submontane grassland after the cessation of intensive fertilization. Preslia.

[33] Hautier Y., Seabloom E.W., Borer E.T., Adler P.B., Harpole W.S., Hillebrand H., Lind E.M., MacDougall A.S., Stevens C.J., Bakker J.D. (2014). Eutrophication weakens stabilizing effects of diversity in natural grasslands. Nature.

[34] Keeley J.E., Keeley M.B., Bond W.J. (1999). Plant species responses along a grazing disturbance gradient in Australian grassland. J. Veg. Sci..

[35] Li J.H., Yang Y.J., Li B.W., Li W.J., Wang G., Knops J.M.H. (2014). Effects of nitrogen and phosphorus fertilization on soil carbon fractions in alpine meadows on the Qinghai-Tibetan Plateau. PLoS ONE.

[36] Morton J.A., Arnillas C.A., Biedermann L., Borer E.T., Brudvig L.A., Buckley Y.M., Cadotte M.W., Davies K., Donohue I., Ebeling A. (2024). Genome size influences plant growth and biodiversity responses to nutrient fertilization in diverse grassland communities. PLoS Biol..

[37] Niu D., Yu M., Xu C., Wang Y., Li C., Yin D., Zuo S., Ren J. (2024). Microbial organic fertilizer improved the physicochemical properties and bacterial communities of degraded soil in the North China Plain. Sustainability.

[38] Wang S., Zhang B., Ma S., Hao J., Zhang L., Guo C., Hong J., Ding H., Zhang Y., Wu Y. (2025). Effects of microbial organic fertilizers on soil microbial communities and physicochemical properties in tobacco cultivation. Front. Environ. Sci..

[39] Wei X., Xie B., Wan C., Song R., Zhong W., Xin S., Song K. (2024). Enhancing soil health and plant growth through microbial fertilizers: Mechanisms, benefits, and sustainable agricultural practices. Agronomy.

[40] Gautam K., Sirohi C., Singh N.R., Thakur Y., Jatav S.S., Rana K., Chitara M., Meena R.P., Singh A.K., Parihar M. (2021). Chapter 1—Microbial biofertilizer: Types, applications, and current challenges for sustainable agricultural production. Biofertilizers.

[41] Murray M.B., Smith R.I., Friend A., Jarvis P.G. (2000). Effect of elevated and varying nutrient application rates on physiology and biomass accumulation of Sitka spruce (*Picea sitchensis*). Tree Physiol..

[42] Yang X., Zhang K., Qi Z., Shaghaleh H., Gao C., Chang T., Zhang J., Hamoud Y.A. (2024). Field examinations on the application of novel biochar-based microbial fertilizer on degraded soils and growth response of flue-cured tobacco (*Nicotiana tabacum* L.). Plants.

[43] Mendoza-Martinez V., Collins S.L., McLaren J.R. (2024). Long-term fertilization increases soil but not plant or microbial N in a Chihuahuan Desert grassland. Biogeosciences.

[44] Da Z.L., Li Y.J., Hu R.M., Yu X.J., Xu C.L., Shi Z.H., Xu J.J. (2024). Effects of different fertilization treatments on vegetation community characteristics and nutritional value of degraded alpine meadows. Grassl. Turf.

[45] Simpson E.H. (1949). Measurement of diversity. Nature.

[46] Shannon C., Weaver W. (1949). The Mathematical Theory of Communication.

[47] Pielou E.C. (1975). Ecological Diversity.

[48] Margalef R. (1958). Information theory in ecology. Gen. Syst..

[49] Bao S.D. (2000). Soil Analysis in Agricultural Chemistry.

[50] Li M., Qi J., Lu X., Zhang T., Yuan Q. (2025). Soil nitrogen prevails in controlling alpine meadow productivity despite Medicago ruthenica reseeding and phosphorus application. Agronomy.

